# Output trends, characteristics, and measurements of three megavoltage radiotherapy linear accelerators

**DOI:** 10.1120/jacmp.v15i4.4783

**Published:** 2014-07-08

**Authors:** Murshed Hossain

**Affiliations:** ^1^ Department of Radiation Oncology Fox Chase Cancer Center Philadelphia PA USA

**Keywords:** linac output, daily output constancy check, 1/f noise, seasonal output variation

## Abstract

The purpose of this study is to characterize and understand the long‐term behavior of the output from megavoltage radiotherapy linear accelerators. Output trends of nine beams from three linear accelerators over a period of more than three years are reported and analyzed. Output, taken during daily warm‐up, forms the basis of this study. The output is measured using devices having ion chambers. These are not calibrated by accredited dosimetry laboratory, but are baseline‐compared against monthly output which is measured using calibrated ion chambers. We consider the output from the daily check devices as it is, and sometimes normalized it by the actual output measured during the monthly calibration of the linacs. The data show noisy quasi‐periodic behavior. The output variation, if normalized by monthly measured “real’ output, is bounded between ± 3%. Beams of different energies from the same linac are correlated with a correlation coefficient as high as 0.97, for one particular linac, and as low as 0.44 for another. These maximum and minimum correlations drop to 0.78 and 0.25 when daily output is normalized by the monthly measurements. These results suggest that the origin of these correlations is both the linacs and the daily output check devices. Beams from different linacs, independent of their energies, have lower correlation coefficient, with a maximum of about 0.50 and a minimum of almost zero. The maximum correlation drops to almost zero if the output is normalized by the monthly measured output. Some scatter plots of pairs of beam output from the same linac show band‐like structures. These structures are blurred when the output is normalized by the monthly calibrated output. Fourier decomposition of the quasi‐periodic output is consistent with a 1/f power law. The output variation appears to come from a distorted normal distribution with a mean of slightly greater than unity. The quasi‐periodic behavior is manifested in the seasonally averaged output, showing annual variability with negative variations in the winter and positive in the summer. This trend is weakened when the daily output is normalized by the monthly calibrated output, indicating that the variation of the periodic component may be intrinsic to both the linacs and the daily measurement devices. Actual linac output was measured monthly. It needs to be adjusted once every three to six months for our tolerance and action levels. If these adjustments are artificially removed, then there is an increase in output of about 2%–4% per year.

PACS numbers: 87.56bd, 87.56Fc, 87.55Qr

## I. INTRODUCTION

Radiotherapy linear accelerators are calibrated to deliver a specific dose under standard conditions following an accepted protocol (for example, AAPM's TG‐51 protocol by Almond et al.^(1)^). The linac output is calibrated to deliver 1.0 cGy per monitor unit (MU) at the depth of maximum output. Beams of photons or electrons pass through a monitor chamber located in the linac head which turns off the beam once the prescribed MU is delivered. The clinical outcome of radiotherapy demands that the linac output does not deviate from the calibrated level by more than a few percent. The output is measured monthly by physicists and recalibrated, if necessary, to keep the output within ±1%. The output is also measured daily by the therapists as a constancy check, and patients are treated if it is within certain tolerance levels. If the output is beyond the tolerance, the linac is recalibrated. In our institution, patient treatment may continue if output is measured to be within ±3% of baseline, in accordance with AAPM TG‐40 recommendations.^(2)^ If output is measured to be between ±3%−5%, patient treatments may continue but output is verified by a physicist at the earliest convenience. Recalibration may then be performed, if needed, and output is brought back to an acceptable level. If the output is measured to be greater than ±5%, patient treatment is halted and recalibration is performed before the treatment continues. Three types of action levels were suggested in AAPM TG‐142 report.^(3)^

Short‐ and long‐term trends can help us understand the intrinsic and controlled change of linac beam output.^4^, ^5^, ^6^ The frequency and action level can be modified based on the results of these type of studies, along with comprehensive statistical analysis of the type done by Sanghangthum et al.^(7)^

## II. MATERIALS AND METHODS

Four oldest linacs in our institution are manufactured by Varian (Varian Oncology, Palo Alto, CA). Each linac gets replaced in about seven to ten years' time. The output for three of the four oldest linacs is analyzed in this work. The fourth one is identical to the third one, both in age and behavior; thus, is not included in this study. The three linacs included are a Varian Trilogy linac (internally designated as T3) and two Varian iX linacs (internally designated as V2 and iX5). All three linacs have dual‐energy photon beams (6 MV and 10 MV on the T3 and the iX5, and 6 MV and 15 MV on the V2) and multiple electron beams (6, 9, 12, 15, 18, and 22 MeV on the T3, and 6, 9, 12, 16, and 20 MeV on the V2 and the iX5). All three of these linacs have sealed Kapton monitor chambers (DuPont, Wilmington, DE). Both photon energies and a single electron energy (9 MeV) beams are analyzed in this study. T3 is about six years old (by the time this manuscript is written, T3 is already decommissioned), V2 is about five years old, and iX5 is a little over four years old. The latest 3.5 years of data are reported. The actual dates of measurements are included in the time axis of various figures presenting time histories. The earlier data are excluded because of unavailability of monthly calibration reports in electronic format. The linacs are calibrated using the AAPM TG‐51 protocol^(1)^ annually to give 1 cGy/MU in water under standard conditions. Output is adjusted monthly by a designated physicist if the output drifts by more than 1.0%. The output constancy checks are performed daily (working days only) by a designated therapist or a service engineer. The daily output is measured with a Keithly ion chamber tracker (Keithly Instruments, Cleveland, OH) on T3, and with a Sun Nuclear Daily QA3 (Sun Nuclear Corporation, Melbourne, FL) ion chamber devices on V2 and iX5; one device for each linac. All three devices use unsealed (open to air) ion chambers. The temperature and pressure are measured by the QA3 devices automatically, but need to be entered manually for the Tracker. All output measurements are corrected for temperature‐pressure variation. The software for the output constancy devices is Argus Software (Varian, Palo Alto, CA). Only the central axis data are analyzed and reported here. The daily central axis output forms the basic data for this work. We have also used the monthly output measurements for some analysis. The monthly output checks are performed by designated physicists using Solid Water phantoms and electrometers and ion chambers whose calibrations can be traced back to the National Institute of Standards and Testing (NIST). Before the monthly output check, various mechanical and other dosimetry checks are performed, including the beam energy, beam flatness, and beam symmetry. The daily measured output is compared against baselines established during annual and monthly measurements, as needed. The output measured daily is reported as independent measurements and only compared to baselines for determining action level. In one part of the study, the independent daily measured output is renormalized and synchronized with monthly measurements. The renormalization factors are kept constant between two monthly measurements.

## III. RESULTS & DISCUSSION

### A. Gross time variation of daily output

**Figure 1:**
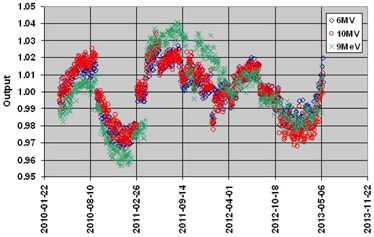
Daily output of 6 MV, 10 MV photon, and 9 MeV electron beams on iX5.

**Figure 2:**
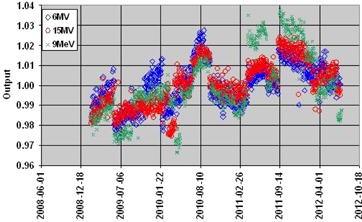
Daily output of 6 MV, 15 MV photon, and 9 MeV electron beams on V2.

**Figure 3:**
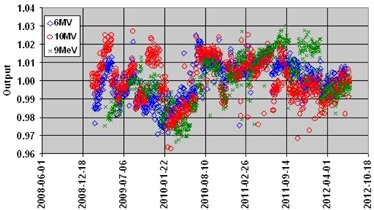
Daily output of 6 MV, 10 MV photon, and 9 MeV electron beams on T3.

The output measured during the daily constancy checks is shown in Figs. 1 to 3, one for each linac. Each figure shows time history of three beams for that linac as measured during the daily constancy check. Since the ion chambers in these devices are not calibrated to give absolute dose, the output of each beam is averaged over the entire period of data gathering, and the average is normalized to unity. Two features can be noted about the output behavior. First, the output of all nine beams in Figs. 1 to 3 shows noisy quasi‐periodic behavior. The term “quasi‐periodic” is meant to indicate a visual recognition of some repetitive behavior, but does not have a clearly defined period. Secondly, the three beams for each linac seem to have some degree of correlation. These characteristics will be discussed below in various subsections. The periodic behavior for the two newer linacs (iX5 and V2, Figs. 1 and 2) is clearer than for the third (T3, Fig. 3). It is noted that the daily QA device for T3 is an older Keithly Tracker with separate electrometer, and the temperature and pressure are entered manually, while the devices used in the other two linacs are Sun Nuclear QA3, and they have built‐in temperature and pressure gauges. Entering the temperature and pressure may be one reason why the T3 data are noisier than with the other two linacs, since manual insertion of temperature and pressure adds a component of human error. Although it is possible that the older device used on T3 could be less stable than the newer devices on the other linacs.

The output presented is not “true” output. The daily output devices are designed to compare daily raw readings against baseline readings set by monthly output measurements. The latter are measured by cross‐calibrated ion chambers traceable to NIST, and represents “true” output of the linacs. However, we have treated the output presented in Figs. 1 to 3 as independent measurements which carry useful information and may be linked to the characteristics of these daily measurement devices, as well as the characteristics of the linac output. Of course, the linac output is adjusted as needed during the monthly calibration. Therefore, the time history presented in Figs. 1 to 3 contains in them characteristics of the daily linac output, as well as any time characteristics of the daily QA devices. These independent output measurements vary slightly more than ±4%. The true output, however, remains within ±3%, as will be presented later by synchronizing the data with monthly measurements. First, we would like to see how the independent measurements presented in Figs. 1 to 3 match up with the monthly measurements.

**Figure 4:**
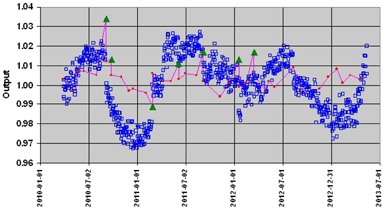
Daily output of 6 MV beam on iX5 (open squares). Superimposed on it are connected solid small circles representing the actual output measured during monthly checks. Large solid triangles are the preadjustment output for the days when it is adjusted.

In Fig. 4, the 6 MV output from iX5 is plotted (open squares) along with the monthly measurements (connected solid circles). When the linac output is adjusted during a monthly measurement, then the output measured before the adjustment is shown in solid large triangles. Each of these points depicted by solid triangles do have a corresponding new adjusted output represented by one of the small solid circle connected by a straight vertical line (since they are taken on the same day, one before the adjustment is made and one after). The overall behavior of the daily output tends to follow the monthly output measurements as they should, but there are some noticeable differences where the daily output shows a tendency of its own. Although the average output for both kinds of measurements is 1.0 or close to it, the raw daily readings sometimes show similar trends as the monthly measurements, but at times the raw readings fall below or above the monthly readings. This is clearly an indication that the daily QA devices have intrinsic time behavior of their own. We will return to this issue later.

**Figure 5:**
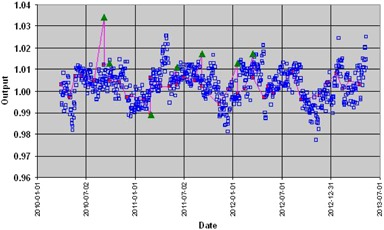
Daily output of 6 MV beam on iX5 (open squares) renormalized by factors to match the actual output after each adjustments. Superimposed on it are connected solid small circles representing the actual output measured during monthly checks. Large solid triangles are the preadjustment output for the days when it is adjusted.

**Figure 6:**
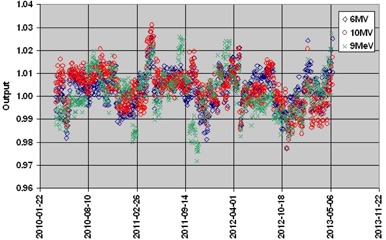
Daily output of 6 MV, 10 MV photon, and 9 MeV electron beams on iX5 renormalized by factors to match the actual output after each adjustments. This is to be contrasted with Fig. 1.

As mentioned earlier, most of the analyses are done using the daily output as independent measurement and only baseline‐compared for constancy check. In Fig. 4, the monthly and daily data are independent of each other. For our next analysis, we modify the daily output by synchronizing it with the monthly output. Daily output is multiplied by a factor to match the monthly output when measured. We note that monthly output is measured only on certain days. The multiplicative factor remains constant until another monthly output is taken. This synchronized output for the 6 MV beam on iX5 is plotted in Fig. 5. Superimposed on it is the monthly output data, shown as connected solid circles. Again the preadjustment output is labeled by solid large triangles on the days the output of the linac is adjusted. Now, the daily output represents as close to the “true” output of the linac as possible without measuring the output with calibrated ion chambers daily. The independent, but synchronized, readings now follow the monthly data more closely than unsynchronized data, as shown in Fig. 4, and lie within ±3%. This is true for all nine beams. The isolated point on 2010‐Sep‐07 in Figs. 4 and 5 must have been an error in the calibration. Figure 6 shows the output synchronized by the monthly measurements for iX5. As a result of independent tendencies of daily QA devices, it is recommended that they be calibrated each time after the monthly output check.

The mean and the standard deviation of all nine beams, both for the raw independent data and synchronized data, are presented in Table 1. The mean of the raw data is normalized to be unity in all cases. For the synchronized data, we see that all nine beams have average values slightly higher than unity. This will be linked to the monitor chamber behavior discussed later in Results section F, where the effect of output calibration is artificially removed, resulting in a tendency of increasing “output” over time.

The quasi‐periodic behavior seen in the raw data (Figs. 1 to 3) reduced to a great extent after synchronizing (for example, in Fig. 6 for iX5). This will be evident in the reduction of spectral power for the few low frequency modes, as presented in the Results section C. The sources of the time variation are unknown, but correlations of various beams may be suggestive. It is noted that no major repair with dosimetric significance, like replacing a magnetron, was recorded for the study period.

**Table 1:** Mean and standard deviation of output and the number of output adjustments performed by physicists during the period of study

	*Raw Data From Daily Output Check*		*Data Reprocessed by Synchronizing with Monthly QA*		*Number of times Linac output is adjusted*
*Linac Beam*	*Mean* ^a^	*St Dev*	*Mean*	*St Dev*	
V2 6 MV	1.000	0.010	1.001	0.008	7
V2 15 MV	1.000	0.011	1.002	0.008	6
V2 9 MeV	1.000	0.016	1.001	0.008	10
T3 6 MV	1.000	0.010	1.003	0.009	11
T3 10 MV	1.000	0.011	1.004	0.009	13
T3 9 MeV	1.000	0.014	1.001	0.009	8
iX5 6 MV	1.000	0.014	1.003	0.007	7
iX5 10 MV	1.000	0.016	1.003	0.008	7
iX5 9 MeV	1.000	0.021	1.001	0.009	10

a The mean is normalized to be unity.

### B. Output correlation of different beams

The output from the daily constancy checks for the three beams for iX5, V2, and T3 shown in Figs. 1 to 3 shows a general trend that all beams of each linac follow each other well, most strikingly for iX5 and less strikingly for T3. To see these correlations more clearly and quantitatively, we show scatter plot of output pairs and compute pair‐wise correlation coefficients.

**Figure 7:**
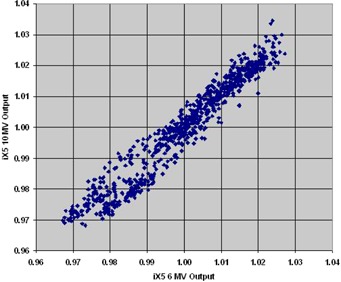
Scatter plot of 6 MV and 10 MV outputs from iX5. The output of these two beams is highly correlated and exhibits a band‐like structure.

**Figure 8:**
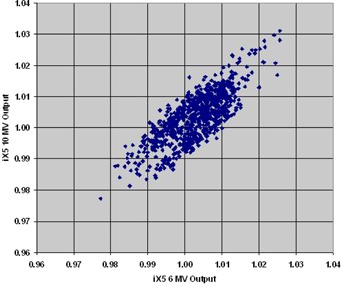
Scatter plot of 6 MV and 10 MV outputs from iX5 renormalized by factors to match the actual output after each adjustments. The output of these two beams remains highly correlated after renormalization.

Figure 7 is a scatter plot of the output for 6 MV and 10 MV beams from iX5. It shows a very strong correlation between the output of the two beams from the same linac. The correlation coefficient is 0.97. This strong correlation is somewhat weakened to a value of 0.78 when the output is normalized by the monthly calibration output. The scatter plot for this case is shown in Fig. 8. The correlation between various beams of the same linac is generally high. The correlation coefficients are 0.83 and 0.80 for 6 MV & 9 MeV and for 10 MV and 9 MeV on iX5, respectively. Likewise, the correlation coefficient between 6 MV and 15 MV beams from V2 is 0.82, while the correlation coefficients between 6 MV and 9 MeV and between 15 MV and 9 MeV are 0.72 and 0.79, respectively, for V2. The lowest correlation between different beams from the same linac is for T3. This is expected, since T3 output has the highest uncertainty due to the older tracker requiring manual temperature and pressure entry.

**Figure 9:**
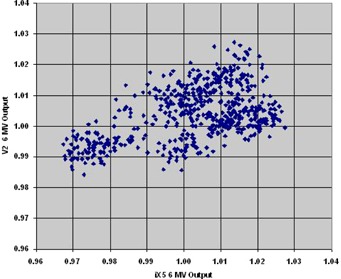
Scatter plot of 6 MV beams from iX5 and V2. The output of these two same energy beams from two different linacs shows a weaker correlation than the beams of the same linac in Figs. 7 and 8.

**Figure 10:**
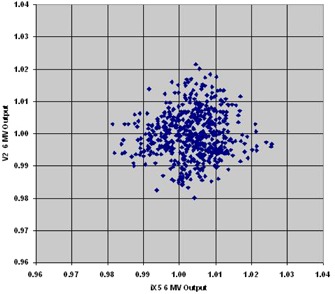
Scatter plot of 6 MV beams from iX5 and V2 renormalized by factors to match the actual output after each adjustments. The weak correlation seen in the raw output of these two beams seen in Fig. 9 has completely disappeared after renormalization.

In contrast, the correlation coefficient between the same energy beams, but from different linacs, is lower, although all beams of all three linacs show quasi‐periodic behavior. The maximum correlation coefficient for such cross‐linac beams is near 0.5. Figure 9 is a scatter graph of 6 MV output from iX5 and V2. The correlation coefficient is 0.50. The correlation disappears completely when the output is normalized by the monthly measured values, as shown in Fig. 10.

The origin of the output variation causing larger correlation coefficients for the two beams from the same linac and a weaker correlation between the same energy, but different linac, cannot be solely attributed to either the linac or the measuring device. This is because we do not measure the daily output of different linacs with the same morning check device. Each linac has its own distinct morning output check device.

The band‐like structures in the scatter graphs might be related to the bimodal or multimodal electronic responses, perhaps caused by branching of electronic circuits.^8^, ^9^ Bifurcations can be caused by discrete values of parameter‐like voltage representing pulse repetition rate or other parameters. More research is needed to understand these band‐like structures — although it might be difficult to have control over various components of the linac in a clinical setting.

### C. Fourier power spectra of time series of output

**Figure 11:**
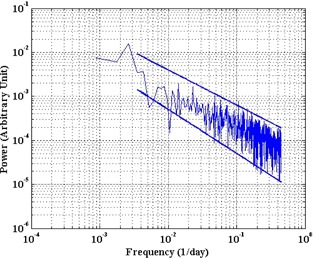
Fourier power spectrum of the time series for 6 MV beam output from iX5. The outer thick solid lines correspond to 1/f^n^ power law with n=1.0 and 0.8. The highest peak corresponds roughly to a period of one year.

Time histories presented in Figs. 1 to 3 show a noisy quasi‐periodic behavior. Similar quasi‐periodic behavior has also been reported by Luketina & Gregg.^(4)^ Although they have not described them as such, but have fitted linear curves for short time segments. The short term data shown in Figs. 1 to 3 can also be fitted with linear curves, but it is not done here. The power spectra of the whole time series may reveal some other characteristics of these variations. Time series representing output as functions of time can be Fourier transformed into functions of frequency. The square of the amplitude of the transformed functions represent power spectra. MATLAB (MathWorks, Inc., Natick, MA) built‐in function “fft” is used for Fourier transforms. Figure 11 shows the power spectrum of 6 MV output on iX5. Power spectra of other beams also qualitatively look similar. There are two noticeable features in the spectrum. First, the spectrum exhibits a power law behavior; second, there is only a weak peak frequency where the most power resides. This is discussed below in detail.

Two thick solid lines are drawn on Fig. 11, corresponding to a 1/f power law. It is customary to call a spectrum of the form 1/f^n^ a 1/f power law for any index n close to unity.^(10)^ The two thick solid lines in Fig. 11 correspond to n=1.0 and n=0.8. The spectrum is consistent with 1/f power law, which is seen in variety of situations in laboratory and nature and considered as a universal power law.^(11)^ There are various theories on how 1/f spectra appear in so many diverse areas of investigations. Hausdorff and Peng^(12)^ have shown for biological systems that, when it is driven by processes having many different scales, it can exhibit an 1/f spectrum. The spectra have been associated with scale invariant distribution of correlation time.^(13)^ There are certainly many sources of noise and driving at different frequencies and amplitude in our case also. Some of these factors include: setup variation in daily constancy check, temperature‐ and humidity‐dependent response of ion chamber and electronics, effects of daily powering on/off of the linac, weekend down time, monthly calibration of the monitor chamber by physicists (when the output is found to be more than 1% away from standard output), annual in‐water output calibration, on/off switching of air‐conditioning unit, and electric power supply variation. The presence of perturbations in many different time scales may be the reason for having 1/f spectrum in our case also.

There seems to be a tendency to flatten the power spectrum shown in Fig. 11 at intermediate frequencies between 0.01 and 0.03, corresponding to a period of about three months and one month, respectively. This may be an indication that there is a random white noise type component in these frequencies with perhaps a flat spectrum which makes the 1/f spectrum a bit shallower at these frequencies.

In the power spectra of time histories of the linac output, there is only a weak peak at 0.0027 1/day, corresponding to roughly a period of one year (as shown in Fig. 11). In the time series, there is an indication of eight to 12 month periodicity. With only 3.5 years of data, we do not have enough number of periods to see a strong peak in the spectrum. We do not have data for long enough time to see a significant bending down of the spectrum at lower frequencies.

**Figure 12:**
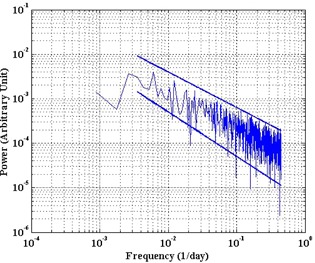
Fourier power spectrum of the time series for 6 MV beam output from iX5 renormalized by factors to match the actual output after each adjustments. The outer thick solid lines correspond to 1/f^n^ power law with n=1.0 and 0.8. The highest peak corresponds to a period of 163 days. The second highest peak is at about one year.

The power spectrum for the output when normalized by the monthly measurements is presented in Fig. 12 for the same energy and linac as in Fig. 11 (iX5, 6 MV). It appears that the power from a few low‐frequency modes in Fig. 12 have reduced relative to Fig. 11. Now the dominant peak is at frequency of 0.006 1/day, corresponding to 163 days, followed by the second peak at about 0.00271/day, corresponding to a period of one year. The peak corresponding to one year was the highest peak in Fig. 11. Please note that the 6 MV output on iX5 was calibrated seven times during the period of study, giving an average of roughly 163 days between calibrations. This period has the highest power in Fig. 12 when the output is renormalized by monthly measurements. This behavior is also seen for other beams.

### D. Frequency distribution of output

**Figure 13:**
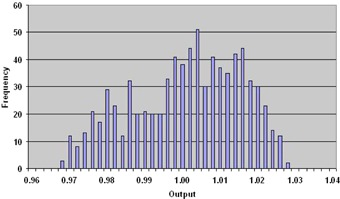
Histogram of distribution of output for 6 MV beam on iX5. The distribution does not appear to be a normal distribution.

**Figure 14:**
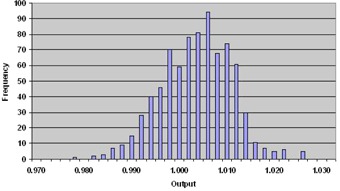
Histogram of distribution of output for 6 MV beam on iX5 renormalized by factors to match the actual output after each adjustments. This distribution is much closer to a normal distribution, with a median value of slightly more than unity.

It is possible to predict how long would it take for the output to deviate from the standard by a given amount if the probability distribution is known. Schultheiss et al.(14) have used a normal distribution to perform such analysis. In Fig. 13, distributions are shown for 6 MV output for the iX5. The distribution does not look like normal. It is possible that it is a normal distribution, but somehow distorted. It is also conceivable that the distribution is a sum of bimodal or multimodal normal distributions. There might be a curious connection between the discrete stripes in the correlation graphs in Figs. 7 and 8 and multimodal distributions in Fig. 13, each stripe with separate normal‐like distribution. A stripe in the scatter plot indicates strong correlation of that part of the data, which is likely to have come from a normal distribution. Each of the different stripes in the same scatter plot may have separate normal distribution with different mean and variance. In such a case, the total distribution will be a superposition of two normal distributions which will not appear to be normal. Other studies also show a shifted normal distribution and, in some cases, a departure from a normal distribution,^(6)^ which could as well be a superposition of two shifted normal distributions. Once the output is normalized by the monthly measurements, the distribution function looks closer to a normal one, as shown in Fig. 14. It may suggest that the distortion in Fig. 13 may not have its origin in the linac itself, rather in the daily QA device.

### E. Seasonal variation of output

**Figure 15:**
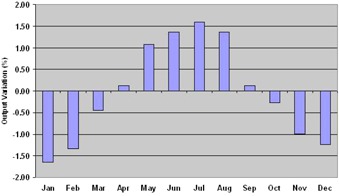
Monthly variation of 6 MV output on iX5. The pattern is consistent with an annual periodicity with positive variation in the summer and negative variation in the winter.

**Figure 16:**
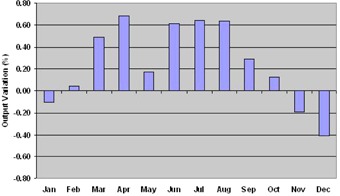
Monthly variation of 6 MV output on iX5 renormalized by factors to match the actual output after each adjustments. The clear annual pattern of Fig. 15 (raw output) is somewhat lost.

The visual indication that the output for three energies for each linac shown in Figs. 1 to 3 is following each other is really a reflection of the fact that all output has a seasonal dependence. To clearly reveal this observation, the output variation of 6 MV beam on iX5 is averaged over each month, Jan. to Dec. The result is displayed as a histogram in Fig. 15. It clearly shows the annual variability with positive output variation in the summer and negative in the winter months. The behavior of other beams is also more or less similar. The output, when normalized by monthly calibrated values, also has remnant seasonal variation, but has broadened, as shown in Fig. 16. The seasonal variation may be related to the variation of humidity and associated electronic response. In particular, resistivity and capacitance, among other things, can be affected by humidity.

### F. Frequency of output adjustments and artificial removal of adjustments made

The output of all beams is found to be bounded by about ±3% when normalized by the monthly calibrated output values. The standard deviation of each beam is listed in Table 1. Deviations range from 0.007 to 0.009. The mean values range from 1.001 to 1.004. The stability is achieved by adjusting the output whenever it is found to have deviated by about ≥1% during the monthly quality assurance checks. In some instances, the output is also checked and adjusted, if needed, in addition to the usual monthly checks. The number of adjustments made during the study period is listed in Table 1. The number of adjustments made for each of the nine beams ranges from six to 13. On average adjustments are made every three to six months. Kapanen et al.^(5)^ suggested that similar treatment quality can be achieved by increasing the output measurement interval and decreasing the action level. It is an interesting policy issue to ponder if the action level for output adjustments is set to a lower value, say, ±0.5% instead of ±1.0%, then could we limit the spread of the output to significantly less than ±3%? If so, can we achieve this by keeping the monthly output checks monthly? Or do we need to do more frequent output checks?

**Figure 17:**
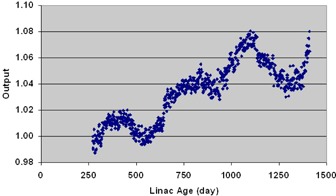
Projected output of 6 MV beam on iX5 if the output adjustments done by the physicists are taken away. The horizontal axis roughly represents the age of the linac.

It is an interesting exercise to pretend that the output adjustments were not done. We can see how the output would have changed over time if the effect of output adjustments is undone. Figure 17 shows such a time series for 6 MV beam on iX5. The output gradually increases, on average. There is about an 8% increase in the output in about 1250 days, giving a value of 2.3% change per year. Please note that the presented data start at a later time then when the linac was installed and used for the first time. We plot the horizontal axis to reflect the actual age of the linac (monitor chamber). Since the linacs under study have sealed monitor chamber, it is conceivable that a slow leak is responsible for the overall increase in the output. The increase could continue for a number of years and then the unadjusted output may saturate. But other investigators have reported that the unadjusted output can increase, in some cases,^4^, ^6^ and decrease in some others.^(6)^ For the cases of increasing output, it is reported to saturate after some years.^(6)^ The increasing and decreasing trends have been attributed to the design differences on the monitor chamber by Kapanen et al.^(15)^ It is not clear if the projected (unadjusted) output in Fig. 17 will continue to increase, saturate, or start decreasing.

## IV. CONCLUSIONS

The output taken during daily constancy check of nine beams from three linear accelerators is presented and analyzed. The data show noisy quasi‐periodic behavior. The output variation is bounded mostly between ±3% and, in worst cases, between ±4%. The time behavior grossly follows the monthly measured output, as seen in Figs. 4 and 5. Beams of different energies from the same linac are correlated with high correlation coefficients. Beams from different linacs, independent of their energies have lower correlation coefficients. The scatter plots of pairs of beams from the same linac show band‐like structures. These may perhaps be linked to branching of electronic circuits^8^, ^9^ based on discrete values of parameter‐like voltage representing pulse repetition rate or other parameters.

The Fourier power spectra of output are consistent with a 1/f power law. This might be due to the presence of factors acting on various time scales. The variations do not appear to come from a normal distribution, rather from a bimodal or multimodal distribution with one peak at negative mean and one at positive mean.

The quasi‐periodic behavior is manifested in the seasonal average output showing annual variability with negative variations in the winter and positive in the summer. The peak power in the spectrum of Fourier‐transformed time series of output occurs at the frequency corresponding to a period of about one year.

Output should be adjusted once every three to six months for our tolerance and action levels. If these adjustments are artificially removed, then there is an increase in output of about 2%–4% per year.

## ACKNOWLEDGMENTS

The author gratefully acknowledges the therapists and the physicists who have generated the data used in this work. The author has benefitted from discussions with Dave Lionis and Jeff Rhoads. Constructive comments by Dr. Charlie Ma and Dr. Robert Price are gratefully acknowledged. The author does not have any conflict of interest. This publication was supported by grant number P30 CA006927 from the National Cancer Institute, NIH. Its contents are solely the responsibility of the author and do not necessarily represent the official views of the National Cancer Institute or the National Institutes of Health.
